# Phytochemical insights and neuro-gut axis modulation of *Asparagus racemosus* (Shatavari) in Postpartum Depression: a mini review with HPTLC-Based justification

**DOI:** 10.3389/fnut.2025.1677952

**Published:** 2025-11-10

**Authors:** Dafini D, Hemavathi Shivapura Krishnarajabhatt, Parvathy Unnikrishnan

**Affiliations:** Department of Stri Roga and Prasuti Tantra (Gynecology and Obstetrics), Amrita School of Ayurveda, Amrita Vishwa Vidyapeetham, Amritapuri, Kollam, India

**Keywords:** *Asparagus racemosus*, Shatavari, microbiota-gut-brain axis, postpartum depression, HPTLC phytochemical profiling, botanical psychobiotics, Ayurveda

## Abstract

Approximately 1 in 8 women experience postpartum depression (PPD), which is a serious public health concern. The adverse effects of antidepressant medications and the stigma attached to receiving mental health care impede the adoption of these approaches. Conventional treatments are seen to be low-risk and give women a feeling of control over their improved physical and emotional well-being. Postpartum depression (PPD) is a complex psychiatric condition increasingly understood through the lens of microbiota-gut-brain axis dysregulation. Recent studies underscore the influence of gut microbiota on neuroendocrine balance, serotonin synthesis, and inflammatory pathways, all crucial factors in the onset and progression of PPD. *Asparagus racemosus* (Shatavari), an Ayurvedic herb traditionally prescribed for women’s reproductive health, is now gaining recognition for its psychobiotic potential. High-Performance Thin-Layer Chromatography (HPTLC) analyses have demonstrated that dry Shatavari contains a significantly richer concentration of bioactive phytoconstituents, such as steroidal saponins and flavonoids, compared to its wet form. These compounds exhibit prebiotic activity, influence microbial composition, and support the modulation of neurotransmitters. This mini review examines the intersection of phytochemical richness in dry *A. racemosus* and its emerging role in microbiota-mediated mood regulation. It highlights its potential as a botanical psychobiotic and proposes directions for future clinical validation within the context of postpartum mental health frameworks.

## 1 Introduction

The postpartum period represents a time of profound physiological and emotional transition. While often celebrated, it is also a window of vulnerability, with postpartum depression (PPD) affecting nearly one in five women globally (1). Traditional explanations for PPD focused largely on hormonal changes, yet emerging evidence highlights the central role of the microbiota-gut- brain axis a complex communication network involving microbial, endocrine, immune, and neural signaling pathways. Gut dysbiosis has been implicated in the onset and severity of PPD, with disruptions in microbial diversity shown to influence serotonin synthesis, inflammation, and hypothalamic–pituitary–adrenal (HPA) axis function (2). Beyond affective disorders, similar mechanisms have also been implicated in neurodegenerative conditions such as Parkinson’s disease, where alterations in microbial composition contribute to neuroinflammation and neurodegeneration. This broader evidence underscores the pivotal role of the gut-brain axis in shaping both mental and neurological health (3). Within this context, botanical psychobiotics, plant-based agents that modulate the gut-brain axis, offer a promising, low-risk intervention for PPD. *Asparagus racemosus* (Shatavari), an Ayurvedic herb traditionally used to support women’s reproductive and mental health, has recently garnered scientific interest for its prebiotic, neuroprotective, adaptogenic and nootropic properties in preclinical settings (4).

These include evidence for modulation of the HPA axis, restoration of disturbed neurotransmitter levels, enhancement of antioxidant enzyme activity, and promotion of neuroplasticity and neurogenesis in brain regions such as the hippocampus and prefrontal cortex (5).

Together, these findings support its potential role as a botanical psychobiotic for mood regulation, especially in contexts of stress and depression.

High-Performance Thin- Layer Chromatography (HPTLC) profiling reveals that dry Shatavari root is notably richer in bioactive compounds compared to its wet counterpart, including steroidal saponins and flavonoids known to affect microbial and neuroendocrine pathways (6). This review explores the intersection of Shatavari’s phytochemical composition and its potential role in modulating the microbiota-gut-brain axis, with a particular focus on postpartum depression.

PPD affects approximately 10–20% of new mothers globally, with increased burden in underserved populations (7). Traditional antidepressant therapies face limitations due to lactation safety concerns and stigma, creating a need for low-risk, effective alternatives. The gut-brain axis, a communication network between intestinal microbiota and the central nervous system has emerged as a critical regulator of emotional health (8). Disruption of gut microbiota has been linked to impaired neuroendocrine signaling and altered neurotransmitter production. Psychobiotics, natural compounds that positively influence mental health through the gut represent a novel therapeutic strategy (9). Among these, *Asparagus racemosus* is gaining recognition for its phytochemical diversity and multifaceted neurobiological potential.

A structured literature search was conducted across PubMed, Scopus, Web of Science, ScienceDirect to identify studies relevant to *Asparagus racemosus* and its role in gut–brain axis modulation. The search used combinations of the following core key terms: “*Asparagus racemosus*,” “Shatavari,” “gut-brain axis,” “microbiota-gut-brain,” “prebiotic,” “psychobiotic,” “short-chain fatty acids,” “neuroinflammation,” “GABA,” “serotonin,” “postpartum depression,” “maternal mental health,” “immune modulation,” “shatavarin,” “saponin,” “flavonoid,” “fructo-oligosaccharide,” and “HPTLC/HPLC.” Boolean operators (AND/OR) were applied to refine retrieval, and no lower year limit was set to ensure inclusion of both classical ethnopharmacological evidence and contemporary mechanistic or clinical research. Only English-language manuscripts were considered. Eligible sources included clinical studies, animal experiments, *in vitro* mechanistic work, phytochemical analyses, and review articles with relevance to neuroendocrine, microbial, immunological, or hormonal pathways. Exclusion criteria comprised agricultural or botanical cultivation papers, non-medicinal reports and articles lacking mechanistic or therapeutic implications. The initial search yielded approximately 430 records, with an additional 35 articles identified through manual reference screening. After removal of duplicates (~70), 360 titles and abstracts were screened. Of these, 110 full texts were assessed for eligibility based on phytopharmacological relevance, mechanistic outcomes, or clinical applicability. A final set of approximately 74 articles were included in the qualitative synthesis.

## 2 The microbiota-gut-brain axis in PPD

Postpartum depression (PPD) is a debilitating mood disorder that affects a significant proportion of women following childbirth, manifesting as persistent sadness, anxiety, irritability, fatigue, and emotional disconnection from the newborn. Traditionally attributed to hormonal imbalances, psychosocial stressors, and genetic vulnerability, recent scientific advances have illuminated a novel and influential player in the onset and progression of PPD within the microbiota-gut-brain (MGB) axis (10). This complex, bidirectional communication network integrates the Central Nervous System (CNS), the enteric nervous system (ENS), the immune system, endocrine pathways, and the diverse community of microbes residing in the gastrointestinal tract (11). Gut microbiota influences the neural, hormonal, and immune signaling through multiple mechanisms, including vagus nerve activation, modulation of the hypothalamic–pituitary–adrenal (HPA) axis, production of neuroactive metabolites like short- chain fatty acids (SCFAs), and regulation of systemic inflammation (12).

During pregnancy, a woman’s gut microbiota undergoes profound changes, characterized by a decline in microbial diversity and a relative increase in pro-inflammatory bacteria, particularly in the third trimester (13). These alterations are influenced by diet, stress, mode of delivery, antibiotic use, and hormonal shifts, and may persist into the postpartum period. Dysbiosis, or the disruption of a healthy microbial balance, has been closely linked to mood disorders, including PPD (14). It results in the compromised synthesis of crucial neurotransmitters such as serotonin, dopamine, and gamma-aminobutyric acid (GABA), all of which are critical for mood regulation (15). Notably, over 90% of the body’s serotonin is synthesized in the gut from the amino acid tryptophan, a process that is heavily influenced by the composition of the gut microbiota (16).

Beneficial microbes such as *Lactobacillus* and *Bifidobacterium* enhance serotonin production, whereas pathogenic or opportunistic bacteria may divert tryptophan metabolism toward kynurenine pathways, producing neurotoxic compounds and lowering serotonin availability (17). Notably, species such as *Clostridium perfringens*, *Escherichia coli*, and *Klebsiella pneumoniae* have been shown to redirect tryptophan metabolism toward kynurenine derivatives, thereby reducing serotonin bioavailability and contributing to neurotoxicity (18).

Additionally, microbial metabolites like SCFAs (butyrate, acetate, propionate) possess anti- inflammatory and neuroprotective properties that help maintain gut barrier integrity, modulate the blood–brain barrier, and influence microglial function within the brain (19). In dysbiotic states, reduced SCFA production and increased gut permeability often referred to as “leaky gut” allow bacterial endotoxins such as lipopolysaccharides (LPS) to enter systemic circulation, triggering a cascade of inflammatory responses (20).

Elevated pro-inflammatory cytokines like interleukin-6 (IL-6), interleukin-1β (IL-1β), and tumor necrosis factor-alpha (TNF-α) have been consistently observed in women with PPD, further supporting the inflammatory hypothesis of depression and the involvement of the MGB axis (21). These cytokines not only affect mood by crossing the blood–brain barrier and altering neurotransmission, but also contribute to the dysregulation of the HPA axis (22). The HPA axis, central to the body’s response to stress, is frequently hyperactivated in depressive states. Under healthy conditions, gut microbes help regulate cortisol levels by modulating feedback mechanisms in the HPA axis (23). However, dysbiosis leads to exaggerated cortisol secretion, chronic stress responses, and impaired resilience, all of which are key features observed in PPD.

Experimental evidence from germ-free animal models has demonstrated the profound influence of the gut microbiota on brain development, stress reactivity, and emotional behavior. These models exhibit heightened anxiety and altered neurotransmitter levels, which normalize upon colonization with healthy microbiota, supporting the essential role of microbes in mood regulation (24).

In humans, several studies have identified distinct alterations in the gut microbiota of women with postpartum depression (PPD). Reductions in beneficial taxa such as *Faecalibacterium prausnitzii*, *Bifidobacterium longum*, and *Lactobacillus* have been reported, accompanied by elevations in pro-inflammatory species including *Clostridium* and *Bacteroides fragilis* (25). Recent high-resolution analyses have begun to resolve specific microbial taxa that associate with increased or decreased risk of PPD. In a large two-sample Mendelian randomization analysis, twelve bacterial taxa were identified as significantly associated with PPD risk: *Veillonellaceae*, *Ruminococcaceae UCG-011*, *Bifidobacterium adolescentis*, *Paraprevotella clara*, *Clostridium leptum*, *Eubacterium siraeum*, and *Coprococcus catus* were inversely associated with PPD risk, whereas *Alphaproteobacteria*, *Roseburia*, FamilyXIII AD3011 group, *Alistipes onderdonkii*, and *Bilophila wadsworthia* showed positive associations with PPD risk (26). These taxon-level associations are consistent with case–control and meta-analytic evidence showing reductions in canonical beneficial genera (for example, *Faecalibacterium*, *Bifidobacterium*, *Lactobacillus*) and relative increases in pro-inflammatory taxa (including members of *Enterobacteriaceae* and *Bacteroides*) in women with PPD (27). Mechanistically, pro-risk taxa such as *Bilophila* and species have been linked to mucosal inflammation and enhanced bile-acid / inflammatory signalling in the gut (28), while other opportunistic organisms can perturb tryptophan metabolism toward kynurenine pathways, reduce serotonin precursor availability, and generate neuroactive metabolites that influence neuroinflammation and HPA axis activity (17, 18). Together, these taxon-specific data strengthen the view that distinct microbial signatures—rather than a nonspecific “dysbiosis”—help define susceptibility to PPD and point to targeted microbiome-based hypotheses for intervention. More recently, another Mendelian randomization study highlighted protective associations for *Prevotellaceae* and *Bifidobacteria*, likely mediated through circulating metabolites such as xanthine and lysophosphatidylinositol, which influence immune and neuroendocrine signaling (29). Taken together, this body of evidence suggests that gut dysbiosis is not only correlated with PPD but may actively contribute to its onset and severity through disrupted microbial composition, impaired metabolite signaling, neuroinflammation, and altered neurotransmitter availability.

Such findings have propelled the exploration of therapeutic strategies targeting the gut microbiota as a means to prevent and manage PPD. Probiotics, defined as live microorganisms that confer health benefits to the host, have shown promise in reducing symptoms of perinatal anxiety and depression (30). Maternal supplementation with *Lactobacillus rhamnosus* HN001 during pregnancy has been associated with a significant reduction in postpartum depression scores (31). Similarly, prebiotics such as fructo- oligo saccharides (FOS) and inulin selectively stimulate the growth of beneficial bacteria and enhance the production of SCFAs and other mood-regulating compounds (32).

## 3 *Asparagus racemosus* and its potent modulating effect on gut microbiota

*Asparagus racemosus* (Shatavari), a revered herb in Ayurveda, possesses significant potential in modulating gut microbiota, making it a promising candidate for gut-brain axis regulation (33). Rich in bioactive compounds such as Shatavarin IV, flavonoids, and fructo- oligo saccharides (FOS), *A. racemosus* exhibits strong prebiotic activity by selectively promoting the growth of beneficial gut bacteria like *Lactobacillus* and *Bifidobacterium* (34). These microbes are key players in maintaining gut homeostasis, producing short-chain fatty acids (SCFAs) such as butyrate, acetate, and propionate that support gut barrier integrity, regulate immune responses, and influence central nervous system function (35).

Studies have shown that dry *A. racemosus* extracts, due to their higher concentration of phytochemicals, exert more pronounced effects in enhancing microbial diversity and richness compared to wet preparations (36). The FOS content serves as a fermentable substrate for commensal bacteria, while the saponins and flavonoids possess anti-inflammatory properties that reduce gut and systemic inflammation (37). By restoring microbial balance, *A. racemosus* indirectly impacts neurotransmitter synthesis, particularly serotonin, produced in the gut, contributing to mood regulation and emotional resilience (38).

Furthermore, its adaptogenic and estrogenic actions complement its microbiota-modulating potential, making it particularly valuable in conditions like postpartum depression (PPD), where gut dysbiosis, hormonal changes, and neuroinflammation intersect (19). Overall, *Asparagus racemosus* acts as a natural synbiotic agent offering both prebiotic nourishment and pharmacological benefits, positioning it as a safe, multi-targeted therapeutic for improving gut health and modulating the microbiota-gut-brain axis (39).

## 4 Prebiotic and synbiotic potential of *Asparagus racemosus*

The prebiotic components of Shatavari, particularly in its dry form, make it a compelling candidate for psychobiotic interventions (40). When used synergistically with probiotic milk, which is known to improve mood and cognitive function, a synbiotic formulation may offer enhanced efficacy (41). Prebiotics nourish these beneficial microbes, boosting the production of short-chain fatty acids (SCFAs) like butyrate, which play vital roles in neurotransmission and gut barrier integrity (42). Through modulation of the kynurenine pathway and reduction in neuroinflammation, these agents may together address both microbial and neurochemical roots of PPD (16).

## 5 Shatavari as a psychobiotic: mechanistic insights

Shatavari influences the gut-brain axis through:

### 5.1 Prebiotic effects

- Contains inulin-type fructo-oligosaccharides (FOS) that selectively nourish probiotic strains like *Lactobacillus* and *Bifidobacterium* (43).
- Promotes SCFA production, especially butyrate, linked with reduced inflammation and improved neuroplasticity (44).

### 5.2 Phytoestrogenic support

- Mimics estrogen, counteracting the postpartum hormonal drop.
- Interacts with microbial *β*-glucuronidase, influencing estrogen recirculation (45, 46).

### 5.3 Anti-inflammatory and adaptogenic action

- Reduces neuroinflammation by suppressing pro-inflammatory cytokines (47).
- Supports HPA axis stability, reducing cortisol dysregulation in PPD (21, 48).

### 5.4 Neurotransmitter modulation

- Enhances serotonin synthesis by increasing available tryptophan (49).
- Reduces kynurenine pathway activation, a depression-linked pathway (50).

**Figure 1:**
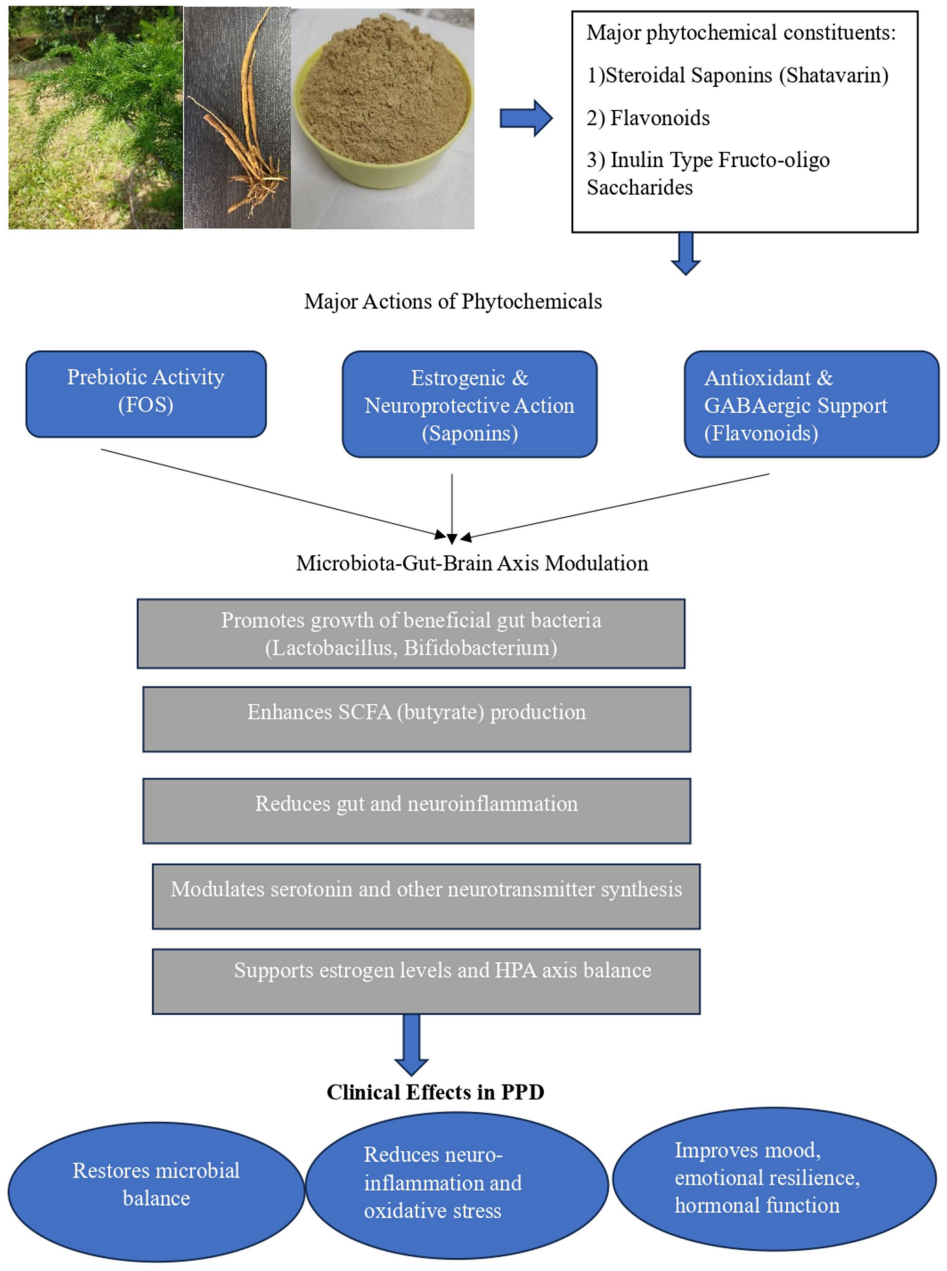
Proposed mechanism of *Asparagus racemosus* (Shatavari) in gut–brain axis modulation and Postpartum Depression. The figure also includes representative images of the *Asparagus racemosus* plant, its tuber, and dried tuber powder, illustrating the medicinal source material associated with neuroprotective, prebiotic, and adaptogenic actions. Original photograph of Asparagus racemosus by Dr. Priyalatha B, Professor, Department of Dravyaguna Vijnana, Amrita School of Ayurveda, Amrita Vishwa Vidyapeetham.

To summarize the current evidence, Table 1 presents the principal phytochemical classes of *A. racemosus*, their effects on gut microbial composition and metabolism, and their corresponding neuroendocrine actions relevant to gut–brain axis modulation. Figure 1 illustrates the proposed mechanism by which *Asparagus racemosus* modulates the gut–brain axis to mitigate pathways implicated in postpartum depression.

**Table 1:** Principal phytochemicals of *Asparagus racemosus* (Shatavari) and reported effects relevant to the gut–brain axis.

Chemical class/extract	Key constituents	Reported effects on gut/microbiota	Reported CNS/neuroendocrine effects relevant to gut–brain axis
Steroidal saponins (4, 33)	Shatavarin I-IV, X	Modulate gut microbial composition; may exert mild surfactant effects that alter microbial ecology; associated with prebiotic-like actions in some phytochemical studies	Estrogenic-like activity, immunomodulation, putative neuroprotective and adaptogenic effects (modulation of stress responses)
Non-digestible oligosaccharides (prebiotic) (4, 33, 34)	Inulin-type fructo-oligosaccharides (FOS)	Fermented by colonic bacteria → ↑ SCFA production (butyrate, acetate, propionate); supports growth of *Bifidobacterium*/ *Lactobacillus*	Indirect CNS effects via SCFAs: improved gut barrier, reduced systemic inflammation, modulation of serotonergic precursors
Polyphenols/flavonoids (4, 6, 33)	Flavonoids (glycosides: quercetin, kaempferol-like, rutin, ferulic acid)	Antimicrobial selectivity, can modulate microbial enzyme activity and favour beneficial taxa; may be metabolized by microbiota to bioactive metabolites May influence microbial fermentation and exert immunomodulatory effects in the gut mucosa (preclinical)	Antioxidant, anti-inflammatory, GABAergic and neuroprotective actions demonstrated in preclinical studies
Complex carbohydrates (35)	Polysaccharides (starch-like fractions)	Contribute fermentable substrate for commensals; support SCFA production and mucosal health	Indirect neuroimmune modulation via reduced gut permeability and systemic inflammation
Sterols (19)	Phytosterols	Minor modulatory effects on microbiota composition reported in phytochemical surveys	Contribute to membrane stability and anti-inflammatory signalling in preclinical work

## 6 *Asparagus racemosus:* clinical forms and combinations

Traditionally, *Asparagus racemosus* (Shatavari) has been used in its fresh tuber form, usually prepared as a milk decoction (*ksheerapaka*). The tubers are gently simmered in milk, which serves both as the base and as a nourishing vehicle (*anupana*), particularly valued in postpartum care and to support lactation. In present-day clinical settings, dried root powder (*churna*) and standardized aqueous or hydroalcoholic extracts; often in the form of capsules or tablets—are used most often as they provide improved availability, stability and dose standardization (51, 52). Phytochemical studies show that careful drying and extraction can enhance the yield of key compounds such as steroidal saponins (shatavarins) and inulin-type fructo-oligosaccharides (FOS). On the other hand, uncontrolled high-heat processing may diminish certain heat-sensitive components. For this reason, standardized extracts are generally preferred when reproducibility is important, as in clinical research. Shatavari can be taken on its own or alongside other botanicals. Classical practice often combined the fresh decoction with ghee (*ghrita*) or incorporated it into compound formulations. It has also been paired with botanicals like *Withania somnifera* (Ashwagandha) and *Emblica officinalis* (Amalaki) to enhance rejuvenation and lactation benefits. Today, clinical use includes both standalone preparations—powder or extract—as well as commercial combination products. Reported clinical trial doses of dried powder or standardized extract typically fall between 500 mg and 2 g per day, though the ideal dose for newer applications such as psychobiotic effects is still under investigation (4).

## 7 *Asparagus racemosus*: HPTLC evidence highlighting key components and the expected outcome at the level of microbiota

### 7.1 Key findings and interpretations

- Higher phytochemical concentration in dry root:

The HPTLC chromatogram under 366 nm revealed consistently higher peak areas and intensities for dry root preparations across Rf values ranging from 0.03 to 0.93. Across all tested application volumes (4 μL, 6 μL, and 8 μL), dry Shatavari demonstrated a greater cumulative area under the curve (AUC), indicating significantly higher concentrations of bioactive compounds compared to wet root preparations (53).

- Distinct and well-resolved phytochemical fingerprints:

Shatavari Ksheera Paka Dry (SKPD) samples showed sharper, denser, and more numerous bands, suggesting clear resolution and separation of diverse bioactive constituents. In contrast, Shatavari Ksheera Paka Wet (SKPW) samples exhibited poor separation and faint bands, indicative of lower phytochemical richness and extractability (54). Enhanced resolution in dry root enhances the reliability of fingerprinting for standardization and pharmacological correlation (55).

- Dominant phytoconstituents identified:
  *Steroidal saponins (notably Shatavarin IV)*: implicated in estrogenic modulation, hormonal balancing, and neuroprotection via inhibition of monoamine oxidase (MAO) (56).
  *Flavonoids*: known to exhibit antioxidant and anti-inflammatory properties, and contribute to modulation of GABAergic activity, which plays a critical role in anxiety and mood regulation (57).
  *Inulin-type oligosaccharides*: act as fermentable prebiotic substrates, selectively nourishing beneficial gut bacteria such as *Lactobacillus* and *Bifidobacterium*, critical for maintaining microbial homeostasis (58).
- Greater microbiota-modulating potential in dry root:

The presence of inulin-type fructo-oligo saccharides (59) in higher abundance highlights the prebiotic strength of dry Shatavari. These non-digestible fibers enhance the abundance of short-chain fatty acid (SCFA)-producing bacteria, thereby influencing intestinal barrier integrity, immune signaling, and the production of neuroactive compounds (60, 61). This places dry Shatavari as a psychobiotic agent with microbiota-mediated modulation of brain function.

- Bioactivity and therapeutic implications:

The superior phytochemical profile of SKPD translates into enhanced biological potential. The combined actions of steroidal saponins, flavonoids, and prebiotics contribute to anti- inflammatory, antioxidant, and neuroprotective effects attributes crucial for managing conditions like postpartum depression (PPD), which involve microbiota dysbiosis, systemic inflammation, and HPA-axis dysregulation (61). The identified phytoconstituents directly or indirectly influence the gut-brain axis via neurotransmitter regulation, immune signaling, and cortisol modulation.

- Enhanced neuroprotective and gut-brain axis impact:

Comparative results demonstrate that SKPD exerts a more pronounced influence on gut-brain axis pathways due to its rich phytochemical reservoir. Higher Shatavarin IV content may support estrogen receptor-mediated neuroplasticity, while flavonoids and SCFA-enhancing prebiotics act synergistically to reduce neuroinflammation and support mental well-being through gut microbial modulation (62).

Table 2 presents the HPTLC profiles of dry and wet roots of *Asparagus racemosus*, highlighting key phytochemical differences. This comparative analysis is included to provide a rationale for linking specific constituents—such as saponins, flavonoids, and fructo-oligosaccharides—to their reported roles in gut microbiome modulation and gut–brain axis regulation.

- *Asparagus racemosus*: safety and tolerability

**Table 2:** HPTLC profiles of wet and dry roots of *Asparagus racemosus* in milk with phytochemical links to gut–brain axis modulation.

Parameter	Dry *A. racemosus* Shatavari Ksheerapaka Dry (SKPD)	Wet *A. racemosus* Shatavari Ksheerapaka Wet (SKPW)	Phytopharmacological relevance & supporting literature
Shatavarin IV content (HPTLC Rf)	High	Moderate	Estrogenic, neuroprotective, MAO-inhibitory activity (56, 67)
Prebiotic potential	Strong (high inulin-type oligosaccharides)	Mild	Supports growth of *Lactobacillus* and *Bifidobacterium*, enhances SCFA production (20, 70)
Antioxidant / Anti-inflammatory action	Higher (flavonoid-rich)	Lower	Reduces neuroinflammation and oxidative stress (66)
Gut–brain axis modulation	Enhanced	Moderate	Modulates GABA, serotonin, and inflammatory pathways (68, 69)
Total bands observed (HPTLC)	Higher in number and intensity	Fewer and faint	Indicates chemical richness and efficient extraction (66)
Major compounds identified	Shatavarin IV, flavonoids, fructooligosaccharides	Trace saponins and flavonoids	Key for hormonal, microbial, and CNS support (66)
Resolution and separation (HPTLC)	Clear, sharp, well-resolved bands	Poor separation and overlapping bands	Ensures precision in phytochemical profiling and standardization (53–55)
Bioactivity implication	Strong neuroprotective, prebiotic, and psychobiotic potential	Comparatively reduced efficacy	Relevant for postpartum depression and gut–brain axis modulation (62)

Clinical and toxicological data indicate that *Asparagus racemosus* is generally well tolerated. Randomized clinical trials in lactating mothers reported no serious adverse events, with occasional mild gastrointestinal complaints. Subchronic animal studies have shown a wide margin of safety, even at high doses, while reviews highlight its safe traditional use. However, due to its phytoestrogenic saponins, caution is suggested in women on concurrent estrogen therapy (62).

## 8 Discussion

Postpartum depression (PPD) is a multifactorial neuropsychiatric condition influenced by hormonal fluctuations, stress, inflammation, and emerging evidence suggests a key role for the microbiota-gut-brain axis in its pathophysiology (63, 64). Targeting this axis with natural agents possessing neuroprotective, hormonal, and microbiota-modulating properties presents a promising therapeutic approach. *Asparagus racemosus* (Shatavari), a well-documented adaptogenic herb in Ayurveda, stands out as a candidate due to its phytochemical richness and diverse biological actions (64). HPTLC-based profiling provides a phytopharmacological basis to support its potential use in PPD management (65).

HPTLC analysis comparing dry and wet preparations of *A. racemosus* roots revealed significantly higher concentrations and diversity of bioactive constituents in the dry form. The dry root powder (SKPD) exhibited stronger chromatographic fingerprints, with sharper and more intense bands across multiple Rf values (0.03–0.93) under UV 366 nm. This indicates a greater presence of steroidal saponins (notably Shatavarin IV), flavonoids, and inulin-type fructo-oligosaccharides compounds known for their neuroprotective, hormonal, and gut- modulating roles. These findings substantiate the dry root’s superior pharmacological potential compared to the wet-processed root (SKPW), which showed fewer bands and poor separation (66).

The steroidal saponins, particularly Shatavarin IV, mimic estrogenic activity, making them highly relevant in PPD, a condition associated with a steep decline in postpartum estrogen (56). Estrogen plays a neurotrophic role in maintaining mood, synaptic plasticity, and stress response regulation (67). Moreover, saponins are implicated in MAO (monoamine oxidase) inhibition, potentially preserving serotonin and dopamine levels, neurotransmitters often disrupted in depressive states (68).

Flavonoids identified in the dry root contribute to antioxidant defense and GABAergic modulation. Since PPD is linked to neuroinflammation and oxidative stress, these compounds offer neuroprotective support by reducing reactive oxygen species and enhancing inhibitory neurotransmission (69). In particular, the modulation of GABA pathways is known to alleviate anxiety and mood disturbances, aligning with the clinical profile of PPD.

Crucially, the presence of inulin-type fructo-oligo saccharides in dry *A. racemosus* enhances its role as a psychobiotic agent. These prebiotic fibres selectively nourish beneficial gut bacteria such as *Lactobacillus* and *Bifidobacterium*, which in turn produce short-chain fatty acids (SCFAs) (70). SCFAs have been shown to reduce systemic inflammation, strengthen gut barrier function, and influence neurochemical synthesis, particularly serotonin, which is predominantly produced in the gut. Thus, phytochemicals synergistically act on both central and enteric pathways of the neuro-gut axis (20).

The enhanced separation and resolution seen in HPTLC further validate the superior chemical integrity of dry root preparations, offering a reproducible and standardized method for quality control in psychopharmacological applications (71). These phytochemical insights justify the preferential use of dry *A. racemosus* root in therapeutic formulations targeting the gut-brain axis, particularly in sensitive populations such as postpartum mothers. The integration of HPTLC data with pharmacodynamic understanding underscores *Asparagus racemosus* (Shatavari) (72) as a potent neuro-gut modulator. Its phytochemical constituents target hormonal, inflammatory, and microbial dysregulations central to postpartum depression, positioning it as a promising plant-based intervention in integrative neuropsychiatry (56).

## 9 Conclusion

With growing recognition of the microbiota-gut-brain axis in postpartum depression, *Asparagus racemosus* emerges as a powerful herb, offering multidimensional support for conventional treatments. HPTLC profiling reveals a phytochemical goldmine rich in Shatavarin IV, flavonoids, and prebiotic oligosaccharides, which directly influences mood, hormone balance, and gut microbial health. Acting as a natural psychobiotic, *Asparagus racemosus* bridges traditional wisdom and modern neuroscience, positioning itself as a safe, holistic, and evidence-backed intervention for PPD. Its ability to nourish both the mind and the microbiome offers a compelling path forward in integrative postpartum care one rooted in nature, validated by science. Future research should prioritize well-designed randomized trials in postpartum women, standardized dry vs. fresh root comparisons, pharmacokinetic and safety studies, and integrated metagenomic–metabolomic approaches to map microbiota–metabolite–host pathways.
